# How to explain exercise-induced phenotype from molecular data: rethink and reconstruction based on AMPK and mTOR signaling

**DOI:** 10.1186/2193-1801-2-693

**Published:** 2013-12-28

**Authors:** Zhengtang Qi, Xiaofeng Zhai, Shuzhe Ding

**Affiliations:** Key Laboratory of Adolescent Health Assessment and Exercise Intervention, Ministry of Education, East China Normal University, Shanghai, 200241 China; College of Physical Education and Health, East China Normal University, Shanghai, 200241 China; Department of Traditional Chinese Medicine, Changhai Hospital, Shanghai, 200438 China

**Keywords:** Exercise, Phenotype, Endurance, Resistance, Skeletal muscle, AMPK, mTOR

## Abstract

During endurance and resistance exercise training, AMPK and mTOR signaling were known as selective pathways implicating the differentiation of exercise-induced phenotype in skeletal muscle. Among the previous studies, however, the differences in exercise protocol, the individuality and the genetic heterogeneity within species make it difficult to reach a consistent conclusion in the roles of AMPK and mTOR signaling. In this review, we aim not to reanalyze the previous articles and present the research progress of AMPK and mTOR signaling in exercise, but to propose an abstract general hypothesis for exercise-induced phenotype. Generally, exercise- induced skeletal muscle phenotype is independent of one and a few genes, proteins and signaling pathways. Convergent adaptation will better summarize the specificity of skeletal muscle phenotype in response to a single mode of exercise. Backward adaptation will open a new concept to illustrate the process of exercise-induced adaptation, such as mitochondrial quality control and muscle mass homeostasis.

Adaptability is essential for survival of lives. Exercise-induced physiological adaptation is often demonstrated by changes in molecules, cells, and organ systems. In skeletal muscle, endurance exercise often increases mitochondrial content and type I myofibers and induces a switch of myofibers from type 2x to type 2a. Resistance exercise often increases muscle protein synthesis and muscle size. All of these changes based on gene expression profile are summarized as exercise-induced phenotype (Figure 1). Endurance and resistance exercise represent extremes on exercise-induced adaptation and produce markedly different phenotypes that are mediated by a complex interplay between AMP-activated kinase (AMPK) and mammalian target of rapamycin (mTOR) signaling (Egan and Zierath, 2013). Exercise physiologists provided a large number of publications to describe the exercise-induced phenotype, they engaged in dissecting the molecular pathways in order to explain: how does human acquire aerobic endurance and muscle strength from endurance and resistance exercise? What gene or protein is important and indispensable for exercise-induced phenotype? With the increase of the publications in this field, it is more and more difficult to guarantee the consistency and validity of exercise-induced phenotype in different studies. How to summarize these molecular evidences and make a story is important for exercise physiologist to understand exercise-induced phenotype.

**Figure 1:**
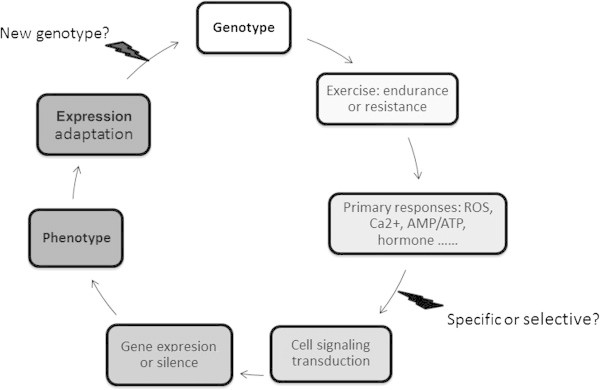
**General schematic for exercise-induced adaptation in physiology: from genotype to phenotype.** Based on a primary genotype, a multiple of signaling pathways are ready to be selected for exercise adaptation. The changes in ROS (Reactive Oxygen Species) level, Ca2+ uptake and release, AMP/ATP ratio and hormone release widely activate or deactivate the downstream pathways during exercise. In response to a single bout of exercise, the underlying pathway is not specific and selective. Repetition of stimulation cycles of exercise leads to an amplification of the cell signaling and thus to the changes in gene expression profile, i.e. exercise-induced phenotype.

## Molecules and genes determine exercise-induced phenotype: really?

Booth and Laye (2009) claimed that a misunderstanding of physiology led to incomplete or wrong functional designations of genes in some cases (Booth and Laye, 2009). Normal physiology should define not only the processes in a non-stressed state but also the capacity of the organism to respond to stressors that disrupt homeostasis. Genes are always selectively activated or deactivated for survival and successful response to stresses. Physical exercise, as a stressor, is used to better demonstrate the complete function of some genes. Gene knockout (KO) or knockdown to restrict its roles in unstressed state may make the organism deprived of the optimal response in stressful conditions. Probably, the animal with gene modification selects the alternative pathway that is enough to respond to exercise stress. In most studies, gene modification failed to change the effects of exercise on muscle mass (Hamilton et al., 2010;Matheny et al., 2009), muscle-fiber switch (Geng et al., 2010;Zechner et al., 2010), mitochondrial biogenesis (Philp et al., 2011;Saleem et al., 2009), and insulin sensitivity (Lira et al., 2010). These findings suggest a theoretical model of functional compensation between genes. Further, Booth and Laye (2009) denied a popular hypothesis: ‘exercise pill’/‘exercise mimetic’. Exercise-induced phenotype in physiology is accomplished by integrating gene, cell, tissue, organ and systems during chronic adaptations to different types of exercise such as resistance and endurance. Thus, exercise-induced phenotype could not be mimicked by a drug or gene modification targeting a single or few molecules. For example, AMPK was both activated by exercise and 5-Aminoimidazole-4- carboxamide1-β-D- ribofuranoside (AICAR). However, the exercise responses differed from those observed with AICAR: plasma fatty acid (FA) and glycerol rose sharply with exercise, whereas FA fell and glycerol was unchanged with AICAR (Rantzau et al., 2008). We always think that exercise capacity is determined by some key genes, because they are found to control cellular metabolism, mitochondrial biogenesis, conversion of muscle fiber type, and protein synthesis, etc. However, knockout or knockdown animals show us that key exercise gene is unlikely to exist. The so-called “exercise-sensitive gene activities” are only the results, not the causes, of exercise adaptation. In other word, without the so-called exercise gene, exercise training still induces cellular phenotype to meet the demands for corresponding exercise or muscle contraction. For example, while PKB/Akt and AMPKα2 activities are essential for Akt substrate of 160 kDa (AS160) phosphorylation during insulin- and AICAR-stimulated glucose uptake in L6 myotubes, neither kinase is indispensable for the effects of muscle contraction on AS160 phosphorylation (Kramer et al., 2006). Interestingly, AS160-Thr(649)Ala knock-in impairs insulin-stimulated glucose uptake in skeletal muscle, rather than contraction and AICAR-stimulated glucose uptake (Ducommun et al., 2012). In conclusion, cellular and molecular phenotype for exercise capacity is always acquired from regular exercise or muscle contraction; it is absurd to use drug and gene modification to mimic exercise-induced phenotype in vivo, while they partly produce functional and molecular phenotype for exercise capacity (Momken et al., 2011;Narkar et al., 2008).

Numerous studies demonstrated that aerobic exercise increased mitochondrial biogenesis in skeletal muscle. Further, these studies aimed to investigate genetic and molecular response to exercise and thus set up the biochemical coupling between exercise and mitochondrial biogenesis. The method is to build exercise protocol of animals and observe the biochemical and molecular changes versus sedentary animals, finally these changes will be integrated to interpret mitochondrial biogenesis. Cell signaling provides a basic framework for understanding the integration of mitochondrial biogenesis and function (Scarpulla, 2008). However, we are facing puzzles and challenges. For example, in-vivo mouse models show that p53 plays an important role in determining both basal aerobic exercise capacity and its improvement by training (Wang et al., 2012). p53 promotes aerobic metabolism and exercise capacity by using different mitochondrial genes and mechanisms in a tissue-specific manner (Park et al., 2009). In p53KO mice, Saleem, A. and colleagues observed diminished mitochondrial content in mixed muscle and lowered PGC-1α protein levels in gastrocnemius muscle. p53-null animals displayed greater fatigability and less endurance than wild-type (WT). However, the adaptive responses in mitochondrial content to running were similar in WT and KO mice (Saleem et al., 2009). These findings suggest that p53 is not required for exercise-induced mitochondrial biogenesis, there must be an alternate mediator that leads to mitochondrial biogenesis. Such important but not required genes or mediators were observed frequently in exercise-induced mitochondrial biogenesis. We suggest that drug treatment or gene modification sometimes makes us misunderstand exercise-induced phenotype within human normal physiology. The reason is that the genetically modified mice are treated equally to the wild-type animals and even human under the condition of exercise. We can’t predict the unknown adverse effects of drug and genetic modification in addition to its ability to enhance exercise capacity. Unfortunately, people believe that molecular modification can exclusively produce exercise endurance and muscle strength, therefore the drug is often abused to promote protein synthesis and red blood cells in games and gymnasiums. Transgenic athletes may also appear in the future. Misunderstanding of exercise-induced phenotype really threatens our health, because the known changes in molecular level are not sufficient for integrated exercise-induced phenotype.

## Resistance and endurance exercise: convergent evolution/adaptation?

Exercise can be classified into two subtypes: endurance and resistance. As we know, exercise- induced phenotype in cell and tissue is determined by exercise protocol. Generally, resistance exercise results in an increase in muscle mass and size, and endurance exercise results in an increase in muscle capillary density, mitochondrial protein, fatty acid-oxidation enzymes, and more metabolically efficient forms of contractile and regulatory proteins (Baar, 2006). However, concurrent training, training for both muscle strength and endurance, suppressed some of the adaptations to strength or endurance training alone in some studies (Bell et al., 2000;Glowacki et al., 2004;Leveritt et al., 1999). There may be a direct molecular blockade hindering the development of the concurrent training phenotype. Therefore, exercise physiologists propose the following pathways: 1) endurance exercise >>> AMPK/PGC-1α signaling>>> mitochondrial biogenesis, this pathway suggests that a selective activation of the AMPK-PGC-1α signaling may explain endurance training adaptations, such as mitochondrial biogenesis (Atherton et al., 2005;Nader, 2006;Reznick and Shulman, 2006;Winder et al., 2006); 2) resistance exercise >>> Akt/TSC2/mTOR signaling >>> cell growth and protein synthesis, this pathway suggests that a specific activation of PKB-TSC2-mTOR cascade may explain some resistance training adaptations, such as increased protein synthesis and muscle growth (Atherton et al., 2005;Baar, 2006;Nader, 2006); 3) endurance exercise >>> AMPK/TSC2/mTOR signaling >>> inhibited cell growth and protein synthesis, this pathway suggests that a negative regulation of mTOR activity byAMPK may explain why endurance exercise damages the effects of resistance exercise in muscle growth (Ng et al., 2012;Reiter et al., 2005). Together, selective activation of AMPK/PGC-1α or Akt/TSC2/mTOR signaling can explain specific adaptations to endurance or resistance training in skeletal muscle. Recently, this assumption is more and more unconvincing. Endurance exercise also enhanced muscle protein synthesis and elevated mTOR signaling in human (Mascher et al., 2011). 10 days of intensified endurance training attenuated AMPK and mTOR signaling, but AMPK and mTOR phosphorylation increased in response to acute endurance exercise (Benziane et al., 2008). On the other hand, strength training increased the protein content of AMPK subunits (α1,β2,γ1), which thus influence metabolism and improve energy homeostasis in trained muscle (Wojtaszewski et al., 2005). AMPK activation and a reduced phosphorylation of 4E-BP1 contribute to the inhibition of muscle protein synthesis during resistance exercise. However, muscle protein synthesis increased in association with an activation of PKB, mTOR, S6K1 and eEF2 by 1-2 h post-exercise (Dreyer et al., 2006). Moreover, endurance and resistance exercise showed a similar time course for Akt-mTOR-S6K phosphorylation during the initial 60-min recovery period after divergent contractile stimuli (Camera et al., 2010). In summary, the hypothesis of selective activation of cell signaling is untenable. The current data strongly indicate that cellular and molecular responses to exercise is very complicated and integrated beyond this hypothesis.

Endurance exercise is defined by higher oxygen uptake, lower muscle contraction force and mitochondria-dependent energy production. Thus, endurance exercise usually improves oxygen utilization and oxidative capacity and increases mitochondrial biogenesis in skeletal muscle. However, these improvements do not depend on the genes controlling mitochondrial biogenesis and oxidation, such as AMPKα (Jorgensen et al., 2005), PGC-1α (Leick et al., 2008) and p53 (Saleem et al., 2009). Lack of PGC-1α reduced expression of Cytc, COXI, and ALAS1 in resting muscle. However, PGC-1α is not required for exercise training-induced increases in ALAS1, COXI, and Cytc expression, showing that factors other than PGC-1α can exert these adaptations (Leick et al., 2008). Also, the improvement of mitochondrial morphology and antioxidant defense in response to endurance exercise occurs independently of PGC-1α function (Geng et al., 2010). For example again, IL-6-deficient mice showed reduced mitochondrial respiration and enzyme activity. However, endurance training still enhanced mitochondrial biogenesis in gastrocnemius muscle. AMPK activation by IL-6 appeared to be dispensable for the mitochondrial biogenic responses to chronic treadmill exercise (Li et al., 2011a). The key protein deacetylase, sirtuin 1 (SIRT1), is a master regulator of mitochondrial biogenesis in skeletal muscle, primarily via its ability to deacetylate and activate PGC-1α (Aquilano et al., 2010;Gurd, 2011;Li et al., 2011b). However, SIRT1 deacetylase activity is not required for deacetylation of PGC-1α or mitochondrial biogenesis in skeletal muscle during exercise (Menzies et al., 2012;Philp et al., 2011). Chronic electrical stimulation of cultured skeletal muscle increased slow myosin heavy chain I (MHCI) and decreased fast MHCII expression at mRNA and protein levels, indicating a fast-to-slow myofiber switch. Calcineurin upregulates MHCI expression via nuclear factor of activated T cells (NFATc1) during fast-to-slow switch of muscle fiber. However, the calcineurin signaling and MHCI gene are activated by repetition of the rapid high-amplitude calcium transients rather than by a sustained elevation of resting Ca2+ concentration. This means that a sustained elevation of calcineurin activity is not available for the formation of slow muscle fiber. In addition, reactive oxygen species (ROS) is supposed to mediate exercise-induced mitochondrial biogenesis (Gomez-Cabrera et al., 2008), because Vitamin E and α-lipoic acid supplementation suppresses skeletal muscle mitochondrial biogenesis after endurance training (Strobel et al., 2011). However, high-dose antioxidant vitamin C supplementation does not prevent acute exercise-induced increases in markers of skeletal muscle mitochondrial biogenesis (Wadley and McConell, 2010). These results suggest us that neither the basal level of ROS and [Ca2+]i nor the presence of some key genes can determine whether exercise activates mitochondrial biogenesis. Repetition of stimulation cycles with proper intensity, like endurance exercise, leads to an amplification of the cell signaling and thus to an increase in mitochondrial content during extended periods of endurance exercise. For endurance exercise, increased oxygen uptake is loaded in cell and consumed only in mitochondria, drug treatment or gene modification to target a single or few molecules unlikely disrupt the predestined outcome: “aerobic exercise >>> increased oxygen uptake >>> increased mitochondrial biogenesis”.

Resistance and strength exercise is characterized by higher muscle contraction force and glycolysis-dependent ATP production. Morphologically, eccentric or concentric strength training leads to differed muscle adaptations. As compared to eccentric strength training, concentric strength training is more likely to lead to pronounced increases in muscle size and muscle hypertrophy (Yasuda et al., 2012). Eccentric exercise induces a greater reduction in muscle force production capability and muscle conduction velocity than concentric exercise (Piitulainen et al., 2011). Eccentric muscle contraction induces greater oxidative stress in skeletal muscle, because migrating inflammatory cells enhanced generation of ROS (Kon et al., 2007). The current findings fail to indicate the molecular and cellular effects of different types of strength training. As compared to endurance exercise, it is very clear that strength training increases protein synthesis and muscle size. Thus, resistance exercise is usually used to improve anaerobic capacity and increase muscle mass and strength. The phosphatase and tensin homologue (PTEN) is critical to activate PI3K/Akt pathway and thus increase muscle mass and growth by altering the level of PI(3,4,5)P(3). Following chronic resistance exercise, however, hypertrophy of skeletal muscles was similar between PTEN(−/−) and PTEN(+/+) animals. Neither PI3K activation nor PTEN is required for overload-induced skeletal muscle growth (Hamilton et al., 2010). Skeletal muscle strength gains from resistance training is independent of circulating insulin-like growth factor (IGF)-I, although upregulation of IGF-I contributes to the growth of muscle that occurs during resistance training (Matheny et al., 2009). The role of mTOR in muscle protein synthesis is ambiguous. Resistance exercise increased muscle protein synthesis and translation of eukaryotic initiation factor 2B (eIF2B) in a mTOR-dependent manner, because this effect was blocked by rapamycin (Kubica et al., 2005). However, the increased anabolic response to resistance exercise is maintained after 4 days of hindlimb unloading, this effect was not blocked by rapamycin (Fluckey et al., 2004). Contrarily, high resistance training frequency augmented inflammatory signaling cascades, the key mediators of anabolic metabolism were strongly suppressed (Coffey et al., 2007). Therefore, skeletal muscle mass may be determined by the timing of resistance exercise-induced overload and recovery. Contrary to our previous hypothesis, resistance exercise also enhances the molecular signaling of mitochondrial biogenesis in human skeletal muscle. Concurrent training is beneficial for the adaptation of muscle oxidative capacity (Wang et al., 2011). Concurrent training-induced acute stimulation of mitochondrial protein synthesis, phosphorylation of Akt and mTOR and PGC-1α expression are equivalent to either single mode (resistance or endurance) (Donges et al., 2012). The protein complex, mTORC1, can also promote the expression of nuclear genes encoding mitochondrial proteins (NUGEMPs) in resting muscle via the interaction of the mTORC1 components and PGC-1α (Carter and Hood, 2012;Cunningham et al., 2007). Disruption of this complex by rapamycin lowered mitochondrial membrane potential, oxygen consumption, and ATP synthetic capacity. mTOR contributes to mitochondrial biogenesis independently of its identified targets (Schieke et al., 2006). Thus, AMPK and mTOR should be highly coordinated, rather than antagonistic, to regulate muscle growth and mitochondrial biogenesis. Generally, AMPK-activated mitochondrial biogenesis and metabolic remodeling during endurance exercise is also a process of muscle protein synthesis depending on mTOR signaling(D’Souza et al., 2007;Ramanathan and Schreiber, 2009), because AMPK signaling is less specific for differentiated exercise (Vissing et al., 2013). If so, what proteins should be synthetized during endurance training, what proteins should be synthetized during resistance training?

**Figure 2:**
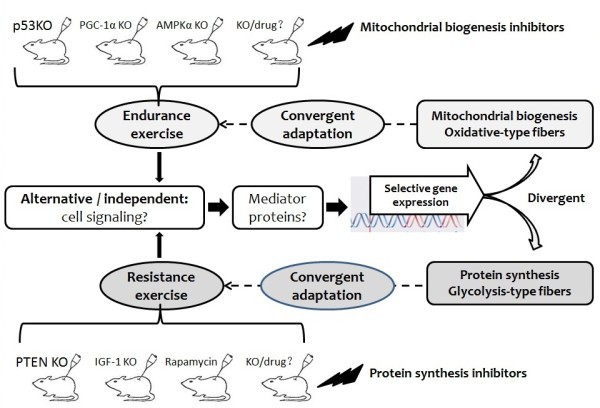
**General hypothesis for convergent adaptation: endurance exercise versus resistance exercise in skeletal muscle.** Skeletal muscle mitochondrial biogenesis is a form of “convergent adaptation” in response to endurance exercise, independent of p53, AMPKalpha and PGC-1alpha. Increased muscle mass and protein synthesis is a form of “convergent adaptation” in response to resistance exercise, independent of PTEN, IGF-1 and rapamycin. Endurance exercise increases gene expression selectively for mitochondrial proteins and enzymes and type I muscle fiber, resistance exercise increases gene expression selectively for muscle growth, anaerobic metabolism and type II muscle fiber. PGC-1α, peroxisome proliferator activated receptor-gamma coactivator 1 alpha; AMPKα, α subunit of AMP- activated kinase complex; PTEN, phosphatase and tensin homologue; IGF-1, insulin-like growth factor 1.

The current findings suggest that mitochondrial biogenesis is a form of “convergent adaptation” in response to endurance exercise, because exercise-induced mitochondrial biogenesis occurs independently of drug and gene modification. Likewise, increased muscle mass and protein synthesis is a form of “convergent adaptation” in response to resistance exercise. Therefore, gene knockout and drugs failed to disrupt mitochondrial biogenesis and muscle growth in many exercise cases. Next, AMPK was acutely activated to increase catabolism during the course of exercise (Lee-Young et al., 2008;McConell et al., 2008), and mTOR was activated to mediate anabolism during recovery (Dreyer et al., 2010;Mascher et al., 2011;Moore et al., 2011). This mode of activation caters to energy demands during and after exercise. We suppose that the molecular events for exercise-induced phenotype mostly occur after exercise and during recovery, thereby leading to specific adaptation to endurance or resistance exercise. Endurance exercise increases gene expression selectively for mitochondrial proteins and enzymes and type I muscle fiber, resistance exercise increases gene expression selectively for muscle growth and anaerobic metabolism and type II muscle fiber (Figure 2). Why? Mounier, R. et al. revealed the diverse functions of the two catalytic isoforms of AMPK, AMPKα1 plays a predominant role in the control of muscle cell size and AMPKα2 mediates muscle metabolic adaptation (Mounier et al., 2011). AMPKα1 is preferentially activated in skeletal muscle following resistance exercise in the absence of metabolic adaptations (McGee et al., 2008). AMPKα2 is usually activated in skeletal muscle to increase mitochondrial biogenesis and metabolic adaptations following endurance exercise (Birk and Wojtaszewski, 2006;Lee-Young et al., 2009;McGee et al., 2003), even if its activity is not essential for increased skeletal muscle fatty acid oxidation (Miura et al., 2009). Recently, Vissing, K. et al. revealed that mTOR signaling is preferentially activated after single-bout strength exercise. However, they found no changes in basal levels of signaling proteins after 10 weeks of endurance or strength training (Vissing et al., 2013). All of these authors tried to found the specificity of the molecular pathway for muscle fiber switch, but the current findings are not convincing because their conclusions can’t stand against the convergent effects of specific exercise, especially when drugs and transgenic mice are used to disrupt the exercise’s effects. Although we have not found the molecular pathway determining muscle fiber switch, the interplay between endurance exercise and resistance exercise is visible, which is closely linked to myofiber-type transformation. Except the adverse effects of endurance exercise on muscle growth, heavy resistance training was found to damage skeletal muscle metabolism. Resistance training significantly decreased insulin-mediated glucose uptake in skeletal muscle (Andersen et al., 2003). Herein, we aim not to ignore the importance of AMPK/PGC-1α for mitochondrial biogenesis and Akt/TSC2/mTOR for protein synthesis in resting skeletal muscle. We suppose that they are only selectable, not indispensable, for exercise-induced skeletal muscle remodeling. In other word, their roles in skeletal muscle adaptation can be replaced by unknown signaling pathways under the stress of single-type exercise (Figure 2).

## From mitochondrial biogenesis to autophagy: backward adaptation?

Increasing findings indicate that exercise adaptation includes not only assimilation such as mitochondrial biogenesis and muscle protein synthesis, and also dissimilation such as protein degradation, autophagy and apoptosis. Previously, more attention was paid to assimilation rather than dissimilation. Really, both of them were concurrently regulated by AMPK and mTOR. Therefore, it is not correct to confine exercise-induced phenotype exclusively to mitochondrial biogenesis and muscle growth, even if the increase in mitochondrial content and muscle mass is the final outcome of various types of exercise. Herein, we define exercise-induced dissimilation as a term of “backward adaptation”. During aging, muscle unloading, and exercise detraining, skeletal muscle backward adaptation is characterized by reduced muscle mass and mitochondrial content versus control group. However, this is not what we hope to discuss in this review. We hope to take mitochondrial quality control for example and set up a novel hypothesis to explain the process of exercise-induced phenotype.

Autophagy refers to a process of degradation of cytosolic components by the lysosome. Autophagy is strongly induced at starvation conditions and during exercise (He et al., 2012;Li et al., 2012;Nair and Klionsky, 2011). The autophagy leads to bulk degradation of proteins, organelles including mitochondria, whose building blocks are recycled for energy supply and the synthesis of components essential for survival (Todde et al., 2009). In unstressed cells, autophagy at basal level is important for the turnover of long-lived proteins and organelles as it can remove exhausted, redundant or unwanted components. Selective elimination of mitochondria by autophagy (i.e. mitophagy), in conjunction with mitochondrial biogenesis, regulates the changes in steady-state mitochondrial number that are required to meet metabolic demand. Recent studies strongly suggest that autophagy including mitophagy plays an important role in maintaining mitochondrial function (Valentin-Vega and Kastan, 2012), skeletal muscle mass (Masiero et al., 2009) and insulin sensitivity (Codogno and Meijer, 2010). Either nutritional or genetic approaches can remove dysfunctional organelles and ameliorate muscular dystrophy via activation of the autophagic flux (Grumati et al., 2011a). Proper activation of autophagy is important for muscle homeostasis during exercise (Grumati et al., 2011b), because the induction of autophagy post- exercise is beneficial to eliminate damaged organelles and maintain cellular homeostasis (Nair and Klionsky, 2011). Although autophagy is a process of proteolysis, disruption of autophagy cannot prevent unloading-induced muscle loss, instead promote the loss of muscle and mitochondrial disorders (Masiero et al., 2009;Sandri, 2010). Autophagy may in part mediate the beneficial effects of exercise in neurodegeneration, adult neurogenesis and improved cognitive function (He et al., 2012). Therefore, we cannot exclusively identify exercise-induced phenotype as the outcome of assimilation.

**Figure 3:**
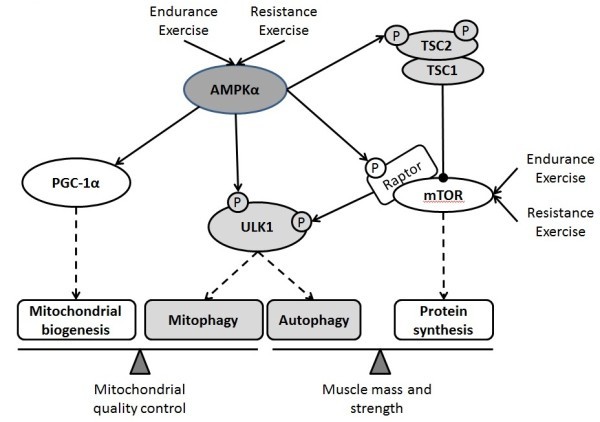
**General hypothesis for exercise-induced backward adaptation: mitochondrial biogenesis versus autophagy.** As an upstream kinase, AMP-activated kinase (AMPK) and mammalian target of rapamycin (mTOR) concurrently control autophagy and protein synthesis in cell, and thus balance mitochondrial biogenesis and mitophagy, muscle growth and autophagy. Both endurance and resistance exercise activate AMPK and mTOR signaling. Autophagic responses to acute and chronic exercise will be so important for exercise-induced phenotype as mitochondrial biogenesis and protein synthesis, this hypothesis is expected to clearly explain: why does AMPK activation during resistance exercise not induce the increase in mitochondrial content? why does mTOR activation during endurance exercise not induce the increase in muscle mass? Upon the activation of AMPK and mTOR, skeletal muscle autophagy is surely involved in the development of exercise-induced phenotype. Ulk1, unc-51-like kinase 1.

AMPK and mTOR, which are considered essential to activate mitochondrial biogenesis and muscle protein synthesis, also interconnect and regulate autophagy (Alers et al., 2012). Generally, AMPK associates with, and phosphorylates, unc-51-like kinase 1 (ULK1), this modification is required for the induction of autophagy after glucose deprivation. When nutrients are plentiful, the mTORC1 complex phosphorylates ULK1, preventing its association and activation by AMPK (Egan et al., 2011). Mitochondria-generated ROS induces autophagy mediated by the AMPK pathway under starvation conditions (Li et al., 2012). During endurance exercise, AMPK triggered a coordinated activation of autophagy, ubiquitin-proteasome pathway and mitochondrial remodeling (Jamart et al., 2012). Exercise increased phosphorylation of AMPK, which stimulates autophagy via suppression of mTOR phosphorylation and protein synthesis, immediately after exercise (Ogura et al., 2011). AMPK both triggers the acute destruction of defective mitochondria through a ULK1-dependent stimulation of mitophagy, as well as stimulates mitochondrial biogenesis through PGC-1α dependent transcription (Mihaylova and Shaw, 2011). In a word, exercise-activated AMPK does not exclusively lead to mitochondrial biogenesis. So does PGC-1α. Expressing PGC-1α in muscle increased the number of lysosomes and autophagosomes (Takikita et al., 2010). These data point to the role of PGC-1α as a master regulator for organelle biogenesis - not only for mitochondria but also for lysosomes and autophagosomes. Contrary to these results, increased PGC-1α levels in skeletal muscle prevented muscle wasting by reducing apoptosis, autophagy, and proteasome degradation (Wenz et al., 2009). Elevated PGC-1α prevented the acceleration of proteolysis induced by starvation and the induction of autophagy via a constitutively active FoxO3. Increasing muscle PGC-1α levels by AICAR prevented loss of mitochondria, although it failed to block loss of muscle mass (Brault et al., 2010). Anyway, PGC-1α regulates not only mitochondrial biogenesis but also autophagic degradation via unknown pathways. The scientists have found a positive correlation between mTOR signaling and muscle mass (Katta et al., 2010;Lang et al., 2012;Winbanks et al., 2012), and a negative correlation between mTOR signaling and autophagy (Alers et al., 2012;Park et al., 2011), suggesting a leading and indispensable role of mTOR signaling in muscle growth. Therefore, mTOR signaling was identified strongly to cause muscle hypertrophy during resistance exercise (Atherton et al., 2005;Mayhew et al., 2009;Wilkinson et al., 2008). If so, why does endurance exercise-induced mTOR signaling lead to mitochondrial biogenesis, rather than muscle hypertrophy? Does autophagy play a role in endurance exercise-induced phenotype? Little is known about the downstream mechanisms of AMPK/mTOR signaling. Based on the current findings, we suppose that AMPK and mTOR concurrently control autophagy and muscle protein synthesis and thus balance mitochondrial biogenesis and mitophagy during exercise adaptation. Autophagic responses to acute and chronic exercise will be so important for exercise-induced phenotype as mitochondrial biogenesis and protein synthesis (Figure 3).

## Conclusion

In summary, AMPK and mTOR signaling are not convincing enough to differentiate the molecular pathways towards exercise-induced phenotype. Among the previous studies, the differences in exercise protocol, the individuality and the genetic heterogeneity within species make it difficult to reach a consistency in conclusion remarks. Lack of adequate appreciation of exercise’s complexities prompts us to propose a novel hypothesis to explain our results at molecular level. In this review, we aim not to summarize all of the previous articles and present the research progress of this field, but to evaluate the paradox among the previous arguments and reconstruct our hypothesis for exercise-induced adaptation. We propose that exercise-induced phenotype is independent of one and a few genes, proteins and signaling pathways. Convergent adaptation will better illustrate the specificity of exercise-induced phenotype under a single mode of exercise. Backward adaptation will open a new thought for exercise-induced adaptation. They will become a target theoretical hypothesis proposed to be confirmed or overturned in the future.
